# Upregulation of miR-4286 and miR-146a-5p in Metastatic Melanoma, Revealed by Multiplex Expression Analysis

**DOI:** 10.3390/cimb48030279

**Published:** 2026-03-05

**Authors:** Iliyan Pochileev, Albena Fakirova, Desislava Tashkova, Aleksandra Gerdgikova, Nevena Ilieva, Denitsa Serteva, Gergana Shalamanova, Hristo Ivanov, Aleksandar Linev, Ivanka Dimova

**Affiliations:** 1 Department of Medical Genetics, Medical University of Sofia, 1431 Sofia, Bulgaria; iliyan.pochileev@gmail.com; 2 Department of Medical Genetics, Medical University of Plovdiv, 4000 Plovdiv, Bulgaria; 3 Clinic of General and Clinical Pathology, Military Medical Academy, 1606 Sofia, Bulgaria; 4 Department of General and Clinical Pathology, Medical University of Plovdiv, 4000 Plovdiv, Bulgaria; 5 Department of Oncodermatology, Oncology Center Plovdiv, 4000 Plovdiv, Bulgaria

**Keywords:** miRNA, metastatic melanoma, BRAF, microRNA biomarkers, NanoString technology, miR-146a-5p, miR-4286

## Abstract

Background: Metastatic melanoma is an extremely aggressive malignancy with limited therapeutic options, despite advances in targeted and immunotherapy. MicroRNAs are key post-transcriptional regulators of gene expression and play a critical role in tumor adaptation, invasion, and metastasis. The aim of our study was to identify dysregulated miRNAs which may serve as novel biomarkers and therapeutic targets. Materials and Methods: The study was conducted on FFPE samples from metastatic melanoma (n = 15), compared to healthy skin tissue (n = 6). BRAF V600E/Ec mutation status was established by Real-Time qPCR. Expression miRNA analysis was performed, using digital counting of 827 miRNAs on the NanoString platform, with data normalization and fold change calculations. Results: Following normalization and quality control metrics, 58 differentially expressed miRNAs were identified in BRAFwt melanoma samples: 6 overexpressed and 52 inderexpressed miRNAs. In BRAFmut melanoma, 37 microRNAs were differentially expressed: 11 overexpressed and 26 underexpressed. Four miRNAs showed elevated expression in both melanoma groups. Among them, miR-146a-5p and miR-4286 demonstrated the highest elevation, especially in BRAFmut tumors. We focused further on their targeted genes. Conclusion: This study demonstrates significant alterations in the miRNA expression profile in metastatic melanoma and highlights the potential of miR-146a-5p and miR-4286 as key regulators of tumor biology.

## 1. Introduction

Metastatic melanoma represents a significant global health challenge due to its aggressive biology and high mortality. According to the most recent data from GLOBOCAN, approximately 331,722 new cases of melanoma were diagnosed worldwide in 2022, and 58,667 patients died from the disease, ranking it among the most common and lethal skin malignancies globally [1].

The etiology of melanoma is multifactorial, but the incidence and severity of the disease are particularly high in regions with elevated ultraviolet radiation exposure, such as Australia, Europe, and North America, where disease rates are significantly higher compared to Africa and Asia [2].

Genetic mutations, including BRAF V600E/Ec, are present in a large proportion of melanoma patients and govern critical molecular pathways involved in tumor proliferation, invasion, and metastatic progression. Despite considerable advances in targeted therapy with BRAF and MEK inhibitors, treatment resistance and clinical progression remain common obstacles to achieving durable responses. This highlights the need for the identification of novel prognostic and predictive molecular biomarkers to improve the personalization of therapeutic strategies [3].

MicroRNAs (miRNAs) are critical regulators of gene expression that coordinate complex signaling networks defining tumor heterogeneity and architecture [4]. They are non-coding RNA molecules which regulate gene expression at the post-transcriptional level by inhibiting translation or inducing degradation of target mRNAs. By selectively modulating the expression of oncogenes and tumor suppressors, miRNAs participate in the control of cell growth, apoptosis, invasion, and interactions with the stromal microenvironment, shaping the structural and functional organization of the tumor [5]. Understanding these regulatory mechanisms provides a unique opportunity to identify novel diagnostic markers and targeted therapeutic strategies, enabling precise recognition of aggressive subpopulations and potential vulnerabilities within the tumor microenvironment [6].

In this context, integrating miRNA profiling into molecular-oncological analysis opens prospects for personalized approaches, directed both at early diagnosis and the development of innovative targeted therapies [7]. These regulators are involved in key biological processes such as cell proliferation, differentiation, apoptosis, epithelial–mesenchymal transition, and immune evasion—all critical for tumor progression and metastatic behavior [8]. Accumulating evidence indicates that miRNA dysregulation contributes to tumor cell adaptation and metastasis formation by coordinating complex signaling networks [9]. The NanoString platform is a modern technology for direct digital counting of nucleic acids without PCR amplification, it minimizes technical artifacts and provides high accuracy and reproducibility of results [10,11].

The platform enables simultaneous multiplex analysis of dozens to hundreds of targets, making it particularly suitable for investigating complex regulatory networks. In the present study, we aimed in studying dysregulated miRNAs in metastatic melanoma with and without BRAF V600E mutation using NanoString gene expression platform, in order to uncover novel molecular targets with potential applications in the diagnosis, prognosis, and personalized treatment of metastatic melanoma. Doing this analysis, we focused on the most prominent overexpressed miRNAs and their molecular and clinical implications.

## 2. Materials and Methods

### 2.1. Patients and Tissue Samples

The present study included formalin-fixed and paraffin-embedded (FFPE) tissue samples from 15 patients diagnosed with metastatic melanoma, as well as 6 control samples of healthy skin tissue. All tumor and control samples were obtained from archival diagnostic material. The study was conducted in accordance with the principles of the Declaration of Helsinki. Written informed consent was obtained from all participants. Patients with metastatic melanoma were stratified into two subgroups based on the mutational status of the BRAF gene: (i) BRAF wild-type (WT) and (ii) BRAFmut (V600E/Ec). This stratification enabled comparative analysis of tumor molecular characteristics according to the presence of a dominant oncogenic driver.

FFPE blocks were selected following histopathological review to confirm diagnosis and assess tumor content. Serial sections were prepared from each block and used for total RNA isolation, optimized for the analysis of small RNA molecules. Control samples from healthy skin were processed using an identical protocol.

### 2.2. DNA and RNA Extraction

Nucleic acids were isolated from FFPE tissue sections or curls (curl input). The recommended amount of starting material ranged from 4 to 8 sections of 5 μm thickness with an approximate tissue area of 25 mm^2^, or 1–6 sections for larger tissue areas of approximately 100 mm^2^. The elution volume of isolated RNA was at least 70 μL, with yield varying depending on sample type, storage conditions, and duration of formalin fixation. Under optimal conditions, 1–2 μg of total RNA was obtained from four FFPE tissue sections (5 μm thickness).

DNA was extracted using TANBead Nucleic Acid Extraction Kit (Taiwan Advanced Nanotech, Taoyuan City, Taiwan) utilizes the nano-magnetic bead based nucleic acid extraction system, together with the exclusive patented whirl stir-mixing automated nucleic acid extraction benchtop instrument (Maelstrom 9600 series, Taiwan Advanced Nanotech, Taoyuan City, Taiwan), which can automatically extract DNA from formalin-fixed paraffin-embedded tissues.

RNA isolation was performed using the TANBead^®^ Nucleic Acid Extraction Kit (M62PA46), designed for the extraction of nucleic acids from formalin-fixed, paraffin-embedded (FFPE) tissues. The deparaffinization process employs mineral oil instead of toxic xylene, making the procedure safer and less damaging to the samples. The resulting RNA was of high quality and suitable for downstream molecular applications, including qPCR, next-generation sequencing (NGS), and NanoString-based expression profiling.

The quality and quantity of isolated RNA were assessed by spectrophotometric measurement (A260/280 and A260/230 ratios) and, when necessary, by RNA integrity analysis to ensure the use of high-quality material for downstream applications.

### 2.3. RT-qPCR Analysis for BRAF Mutational Status

To determine the mutational status of the BRAF gene, quantitative RT-qPCR analysis was performed. Tumor DNA was isolated, and specific primers targeting the BRAF region, which includes the most common oncogenic mutation—V600E/Ec, were used for amplification. PCR reactions were carried out in accordance with the manufacturer’s instructions using real-time quantitative PCR with the in vitro diagnostic Easy^®^ BRAF Kit (Diatech Pharmacogenetics, Jesi, Italy). The assay is intended for the qualitative detection of somatic BRAF codon 600 mutations (V600E/V600E complex, V600K, V600D, and V600R) in genomic DNA isolated from fresh, frozen, or formalin-fixed paraffin-embedded (FFPE) tumor tissue. Each sample was analyzed in triplicate, and no-template controls as well as inter-plate controls were included to assess assay accuracy and reproducibility. PCR data were analyzed using specialized quantitative analysis software, with correction for amplification efficiency and inter-plate variability. Data normalization was performed using reference genes suitable for FFPE material to correct for sample-to-sample variability.

### 2.4. miRNA Expression Analysis

**Figure 1:**
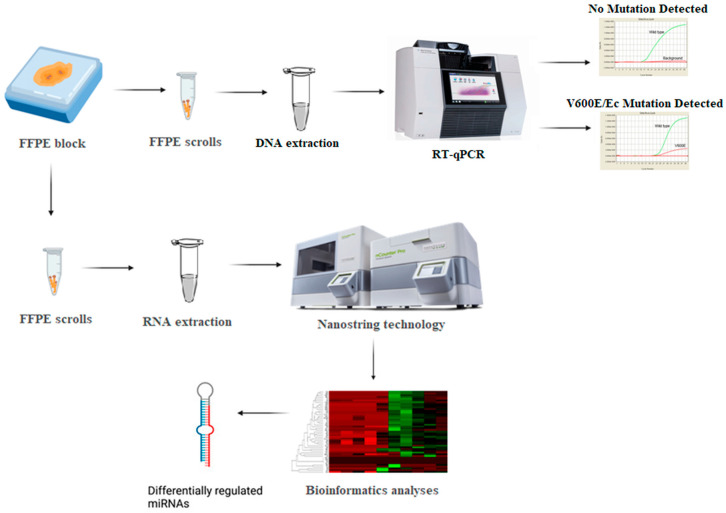
Experimental workflow.

Following total RNA extraction, a miRNA tag ligation reaction was performed, followed by quantitative assessment of the expression of 827 selected miRNAs using digital counting on the NanoString platform using the nCounter^®^ miRNA Expression Assay (NanoString Technologies (Seattle, WA 98109, USA)), which is designed to provide highly sensitive, reproducible, and strongly multiplexed detection of miRNAs across a wide range of expression levels. This assay enables miRNA detection without reverse transcription or amplification, relying instead on molecular barcodes known as nCounter Reporter Probes. The assay is compatible with total RNA isolated from diverse biological sources, including FFPE samples—Figure 1.

The assay protocol includes detailed procedures for miRNA sample preparation and hybridization with the miRNA CodeSet. Post-hybridization processing and data analysis were conducted according to the nCounter^®^ Analysis System User Manual (MAN-C0035) (NanoString Technologies, Seattle, WA, USA), and the Gene Expression Data Analysis Guidelines (MAN-C0011). NanoString technology is based on direct molecular barcoding and digital detection of target molecules using a color-coded probe pair. Each probe pair consists of a Reporter Probe, carrying a fluorescent signal at the 5′ end, and a Capture Probe, bearing biotin at the 3′ end. The color code complexity—comprising four colors arranged across six positions—allows for discrimination and identification of a large number of targets within the same sample during data acquisition. During the overnight hybridization reaction, probe pairs are present in large excess relative to the target nucleic acids, ensuring that each target molecule efficiently hybridizes with its corresponding probe pair. Digital images are processed, barcodes are counted, and the data are exported in comma-separated values (CSV) format. The resulting data can be analyzed using nSolver™ Software version 4.0 or other compatible data analysis tools.

Raw expression data were processed and normalized using the manufacturer’s software, followed by comprehensive bioinformatic analysis, including differential expression assessment, hierarchical clustering, and pathway enrichment. This integrated workflow facilitated the identification of differentially regulated miRNAs, which were further evaluated for their potential functional relevance in tumor biology and as candidate biomarkers for disease progression.

Data normalization and fold change (FC) calculations were performed using nSolver Analysis Software v4.0, ensuring reliable comparison of expression profiles between tumor and control samples, as well as among subgroups stratified according to BRAF mutational status.

### 2.5. Statistical Analysis and Bioinformatic Stringency

To ensure the technical reliability of the microRNA expression profiles, the following statistical framework was implemented using nSolver Analysis Software v4.0 (NanoString Technologies). The analysis followed a multi-tiered approach designed to minimize technical bias and prioritize high-confidence regulatory events:

Dual-Stage Normalization Architecture: We employed a robust normalization strategy to correct for technical and sample-to-sample variability. First, Positive Control Normalization was conducted using the geometric mean of internal synthetic spike-ins to account for platform-associated processing variations; normalization factors were strictly required to fall within the 0.3–3.0 range. Subsequently, CodeSet Content Normalization was performed using the geometric mean of five pre-validated mRNA reference genes (ACTB, B2M, GAPDH, RPL19, and RPLP0) to adjust for differences in RNA input and quality, ensuring a reliable basis for inter-sample comparison.

Stringent Background Filtering (LOD): To mitigate the risk of false-positive findings associated with low-abundance transcripts, we applied a rigorous Limit of Detection (LOD) threshold. The LOD was defined as the mean of the negative control probes plus two standard deviations. MicroRNAs with counts falling below this threshold were manually excluded from the differential analysis. Furthermore, to enhance the robustness of our conclusions, only microRNAs exhibiting expression levels above the LOD in more than 50% of the samples within a study group were included in the final computational workflow.

Differential Expression (DE) Criteria: Quantitative assessment of differential expression was performed by calculating Fold Change (FC) ratios derived from normalized digital counts. We utilized a stringent inclusion cutoff of an absolute FC > 2 to distinguish significant transcriptomic shifts from inherent biological noise.

Significance Testing and Quality Metrics: Statistical significance for the identified differentially expressed microRNAs was determined based on the integrated algorithms of the nCounter^®^ Analysis System, adhering to the MAN-C0011 and MAN-C0035 guidelines. *p*-values were calculated to assess the probability of the observed distribution, with significance thresholds established to identify the most consistently deregulated candidates. Technical assay performance was further validated by ensuring that POS_E control signals (at the 0.5 fM detection limit) and positive ligation controls exceeded background levels and displayed the expected linear trends across all runs.

This systematic statistical framework provides a high level of confidence in the digital quantification of the miRNA landscape, ensuring that the subsequent results regarding tumor-specific dysregulation and shared regulatory axes are supported by rigorous methodology.

### 2.6. Identification of Potential Target Molecules

To identify candidate miRNAs associated with key oncogenic and tumor-suppressor pathways, experimental expression data were integrated with in silico analyses using established databases, including TargetScan, miRTarBase, miRecords, and miRTargetLink 2.0. Putative miRNA regulatory networks were visualized, highlighting key nodes associated with BRAF-related signaling cascades (MAPK/ERK) and tumor-suppressive pathways such as PTEN/PI3K/AKT. These in silico models enabled prioritization of miRNAs that not only exhibited significant differential expression but also demonstrated high potential to regulate critical oncogenic targets and pathways. This integrative approach, combining experimental data with computational predictions, provides a systematic framework for identifying therapeutically relevant miRNAs in metastatic melanoma and establishes a foundation for subsequent functional validation studies.

## 3. Results

### 3.1. Dividing Subgroups of Melanoma According to BRAF Mutational Status

BRAF mutational status was determined by real time PCR analysis. Samples were divided according to BRAF mutational status (WT vs. V600E) in two groups: BRAFmut—n = 7 and BRAFwt—n = 8.

### 3.2. miRNAs Profiling by NanoString nCounter Analysis

Total RNA expression was analyzed using the nCounter^®^ Human miRNA Expression Assay v3 (NanoString Technologies). The assay panel targeted 827 human microRNAs and contained six positive controls, eight negative controls, six ligation controls, and five mRNA reference genes (ACTB, B2M, GAPDH, RPL19, and RPLP0). A volume of 5 μL of concentrated RNA was used as input for the analysis.

Positive control normalization was carried out using the geometric mean, with normalization factors flagged if they fell outside the acceptable range of 0.3–3.0. Codeset content normalization was performed using the reference genes, applying geometric mean normalization and flagging values outside the 0.1–10.0 normalization factor range. Background noise was eliminated by manually excluding microRNAs with counts below the limit of detection (LOD) thresholds, which was defined as the mean of the negative controls plus two standard deviations. Differential expression was assessed by calculating fold changes (FC) using nSolver ratio outputs derived from normalized count data.

The quality control metric for the limit-of-detection (LOD) of the six positive controls evaluates whether the signal obtained from the POS_E control probe, introduced at a concentration of 0.5 fM (considered the system’s detection limit), is significantly greater than the signal from the negative control probes. Under acceptable conditions, POS_E counts are expected to exceed background levels. In the present study, the POS_E probe signal was higher than that of all negative controls.

Each miRNA assay includes six synthetic RNA constructs that function as ligation controls. Three constructs undergo ligation and release a miRNA tag, serving as positive ligation controls, while the remaining three do not undergo ligation and act as negative ligation controls. Under optimal assay performance, the negative ligation controls should produce signals within background levels, whereas the positive ligation controls should generate substantially higher counts, increasing progressively from LIG_POS_C to LIG_POS_A. In this study, all positive ligation control signals exceeded all defined LOD thresholds and displayed the expected increasing trend from Lig_Pos_C to Lig_Pos_A, while all negative ligation controls remained below all LOD thresholds.

Following normalization, in the group of BRAFwt melanoma, 231 microRNAs exceeded the LOD threshold in at least one of the samples, while 39 microRNAs were consistently detected in all samples (including also health skin tissues)—Table 1. We performed the analysis taking miRNAs with detected expression in more than 50% of samples—totally 122 miRNAs.

In the group of BRAFmut melanoma, 275 microRNAs showed signals above the threshold in at least one sample, with 49 showing such expression in all samples—Table 1. Totally, 155 miRNAs had expression above threshold in more than 50% of samples (both tumor and controls) and were included in our analysis.

### 3.3. Differentially Expressed miRNAs in BRAFwt Melanoma

**Figure 2:**
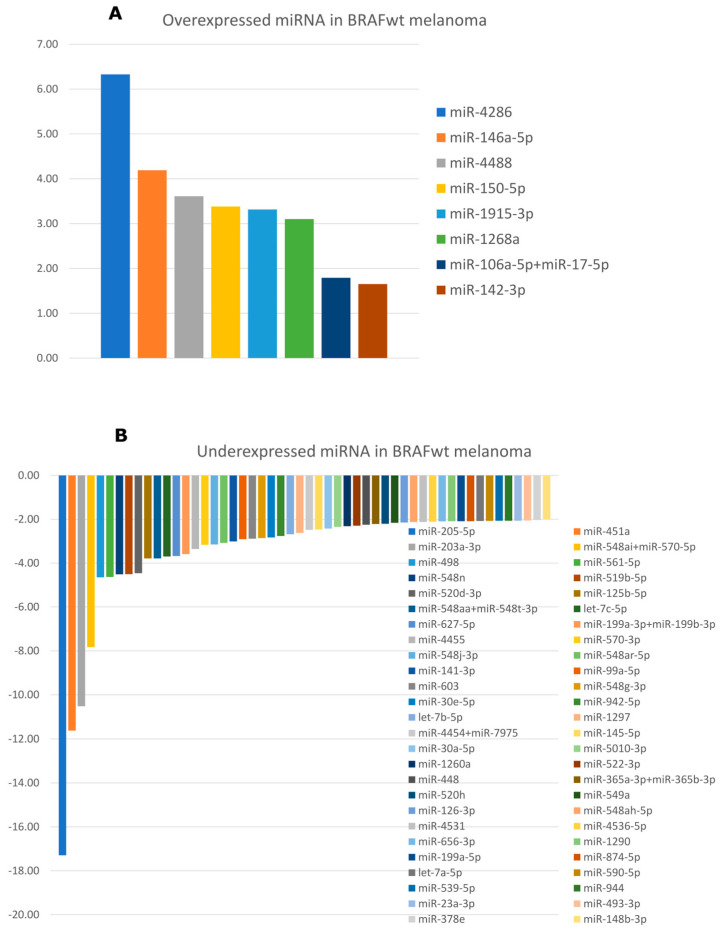
Differentially expressed miRNAs in BRAFwt melanoma: (**A**) overexpressed miRNAs and (**B**) underexpressed miRNAs.

Using nSolver Analysis Software v4.0, 58 differentially expressed miRNAs were identified in the dataset of BRAFwt melanoma samples, compared to health skin controls: 6 overexpressed and 52 inderexpressed miRNAs. Figure 2 represents these miRNAs with differential expression (FC > |±2|). The highly expressed were miR-4286 (FC = 6.33), miR-146a-5p (FC = 4.19) and miR-4488 (FC = 3.61). The lowest expression was detected for miR-205-5p (FC = −17.3), miR-451a (FC = −11.6) and miR-203a-3p (FC = −10.5).

### 3.4. Differentially Expressed miRNAs in BRAFmut Melanoma

**Figure 3:**
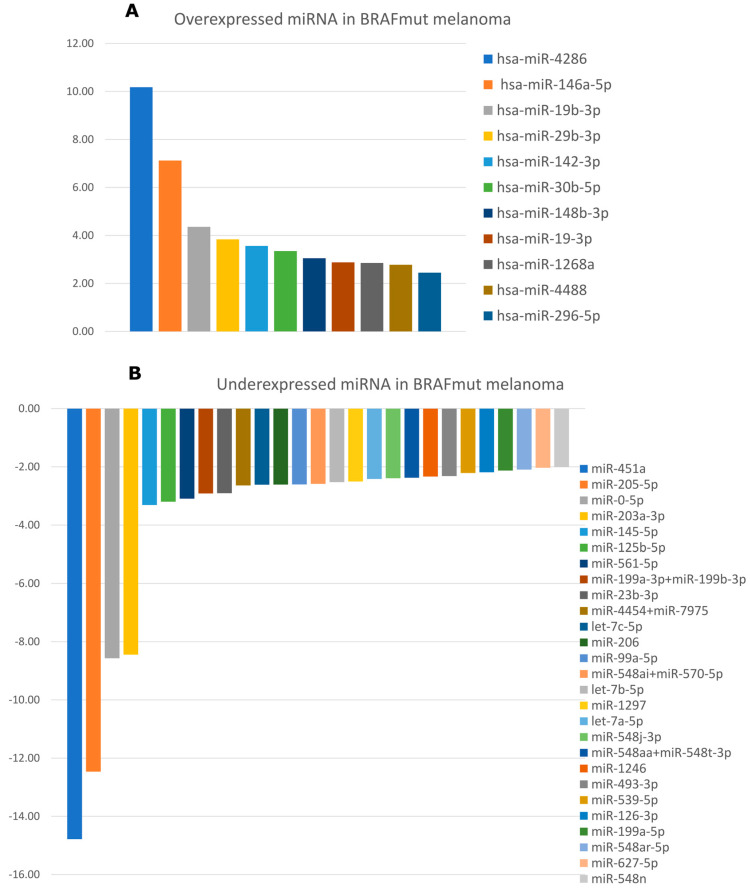
Differentially expressed miRNAs in BRAFmut melanoma: (**A**) overexpressed miRNAs and (**B**) underexpressed miRNAs.

Totally, 37 microRNAs were differentially expressed in BRAF+ melanoma: 11 were overexpressed and 26 were underexpressed—Figure 3. Among the overexpressed miRNAs, the highest expression was detected for miR-4286 (FC = 10.18), miR-146a-5p (FC = 7.12) and miR-19b-3p (FC = 4.4). The lowest expression showed miR-451a (FC = −19.14), miR-205-5p (FC = −16.14) and miR-203a-3p (FC = −12.54).

### 3.5. Common Overexpressed miRNAs in BRAFwt and BRAFmut Melanoma

**Figure 4:**
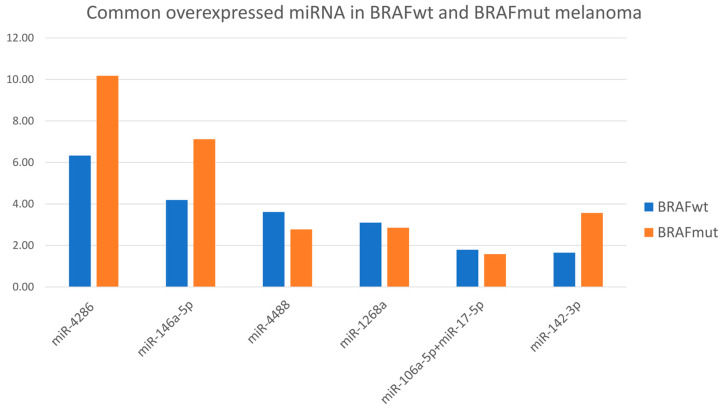
Overexpressed miRNAs in both BRAFwt and BRAFmut melanoma.

We focused on the overexpressed miRNA further in our analysis here. Four miRNAs showed elevated expression in both BRAFwt and BRAFmut melanoma—Figure 4 Among them, miR-146a-5p and miR-4286 demonstrated the highest elevation, especially in BRAFmut tumors—Table 2.

The average expression levels of miR-146a-5p and miR-4286 were significantly elevated in melanoma samples compared to controls. This mean expression profile highlights the consistent deregulation of these two miRNAs and suggests their involvement in key molecular processes, including cytoskeletal remodeling, cell migration, and invasion. Further assessment of the two highly expressed miRNAs, miR-146a-5p and miR-4286, demonstrates distinct expression patterns across sample groups. Both miRNAs showed higher expression in BRAFmut melanoma samples compared to BRAFwt tumors. These results support the role of miR-146a-5p and miR-4286 as candidate regulators of tumor progression and suggest their involvement in pathways related to cell proliferation, migration, and metastatic potential.

### 3.6. Identification of Potential Target Genes for miR-146a-5p and miR-4286

Potential target genes of these miRNAs were determined based on experimentally validated interactions retrieved from miRTarBase and other curated sources integrated within miRTargetLink 2.0. Following filtering with miRTargetLink 2.0, the analysis focused on experimentally validated miRNA–target interactions exhibiting inverse expression patterns. This approach led to the identification of specific targets of differentially expressed miRNAs that are potentially involved in tumor progression and metastatic processes in melanoma patients.

**Figure 5:**
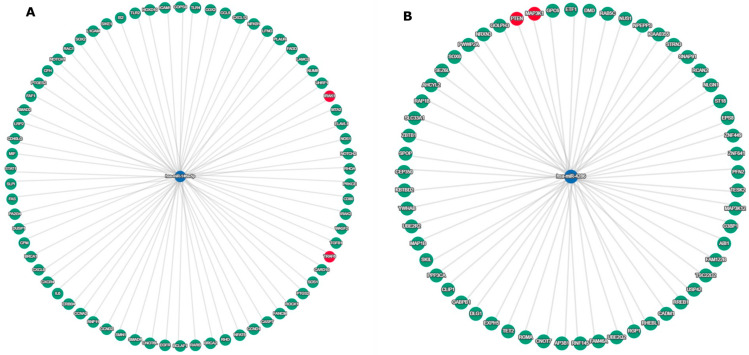
Targeted genes for miR-146a-5p (**A**) and miR-4286 (**B**), retrieved from miRTargetLink 2.0. The highly ranked target genes are marked in red: TRAF6 and IRAK1 for miR-146a-5p and PTEN and MAP3K1 for miR-4286.

There are 488 predicted targets for hsa-miR-146a-5p in miRDB (https://mirdb.org/cgi-bin/search.cgi?searchType=miRNA&full=mirbase&searchBox=MIMAT0000449 (accessed on 19 January 2026)). The highly ranked targets (Target score = 100) and also experimentally validated via luciferase reporter assays are *TRAF6* and *IRAK1*—Figure 5, marked in red. miR-146a-5p most strongly affects the NF-κB inflammatory signaling pathway by directly targeting *IRAK1* and *TRAF6*, acting as a master negative regulator of innate immune responses. Analysis using the TargetLink 2.0 database indicated the highly predicted targets for miR-4286 are *PTEN* and *MAP3K1* (Figure 5, marked in red). However, only a few targets are experimentally validated for this miRNA—*TGFB1*, *TGFBR2*, *RUNX3* and *HDAC3*. Associative analysis further revealed that TGF-β and NF-κB are interconnected, and these two miRNAs may have a role that could be valuable for future studies and targeted therapies in metastatic melanoma.

**Figure 6:**
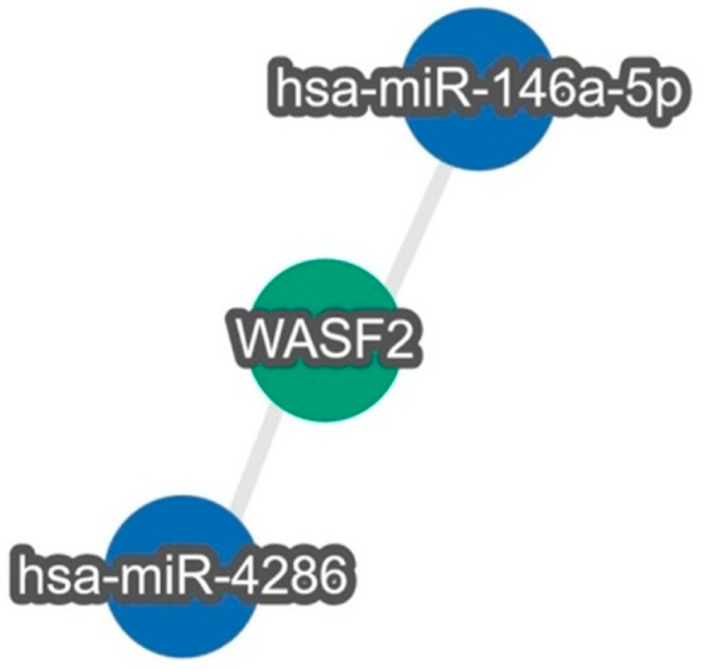
Regulatory relationship between miR-146a-5p and miR-4286 with WASF2 gene, identified using miRTargetLink 2.0. Bioinformatic analysis revealed that both miRNAs share a common target, WASF2, and possess conserved binding sites within the 3′-UTR region of WASF2 mRNA.

Analysis through miRTargetLink 2.0 revealed that both miR-146a-5p and miR-4286 target the WASF2 gene (Figure 6), indicating a shared regulatory mechanism affecting a key component of actin cytoskeleton dynamics. Given the central role of WASF2 (WAVE2) in cell migration, lamellipodia formation, and invasive phenotypes, these findings support the hypothesis that the observed miRNA deregulation contributes to the enhanced metastatic potential of tumor cells.

The dual targeting of WASF2 by two distinct miRNAs suggests fine-tuned, coordinated post-transcriptional regulation, which may be critical for cytoskeletal remodeling and disease progression. In the context of melanoma, particularly BRAF-positive tumors, the miRNA–WASF2 axis likely modulates key signaling pathways associated with invasion and metastasis, representing a potential target for future functional and therapeutic studies.

## 4. Discussion

Using NanoString nCounter platform in our study, we reliably identified the differentially expressed microRNAS in metastatic melanoma, both BRAF-wild type and BRAF-mutant. Here we focused on the most promising overexpressed microRNAs in these tumors—miR-146a-5p and miR-4286.

The first one, miR-146a-5p, as a negative regulator of NF-κB, suppresses excessive immune activity, but in the context of melanoma survival, this “dampened inflammation” supports tumor immune evasion [12]. It is one of the best-characterized regulators of the immune and inflammatory response. This miRNA is part of a classic negative feedback loop of NF-κB, in which, upon activation of inflammatory signals (e.g., through TLR/IL-1 receptor pathways), NF-κB induces transcription of miR-146a-5p [13]. Subsequently, miR-146a-5p suppresses key nodal proteins such as IRAK1, SMAD4, and TRAF6, leading to attenuation of the inflammatory cascade. In melanoma, NF-κB is often constitutively active due to chronic cellular stress, oncogenic signaling, and the influence of the tumor microenvironment. In this context, miR-146a-5p acts as an adaptive modulator that partially “buffers” hyperactive NF-κB signaling, maintaining a lower inflammatory tone. This benefits tumor cells, as the reduced inflammatory signal favors immune evasion [14,15,16].

Increased expression of miR-146a-5p in melanoma is associated with activation of transformational programs, including changes in adhesion properties, reduced E-cadherin, and upregulation of molecules linked to EMT-like phenotypes [17,18]. This facilitates progression toward a more aggressive behavior and metastasis. miR-146a-5p also influences the secretion of key cytokines (e.g., IL-6, IL-8, CCL2), which participate in remodeling the immune microenvironment [19]. Its activity leads to a reduction in antigen-presenting potential and a decreased response from dendritic cells and T cells. Various studies have shown consistently elevated levels of miR-146a-5p both in tumor tissue and circulating exosomes in melanoma patients. This positions it as a candidate for a non-invasive biomarker for early detection, tumor activity assessment, and prognostic stratification.

The second overexpressed microRNA, miR-4286, is less characterized compared to miR-146a-5p, but there is consistent experimental evidence showing increased expression in melanoma cell lines and tumor samples [20,21]. Its mode of action is primarily oncogenic. There is evidence that activated MAPK/ERK signaling increases transcriptional activity on loci regulating miR-4286 [22,23,24]. This leads to overexpression, reflecting the oncogenic needs of BRAFmut tumors. The combination of BRAFmut and high levels of miR-4286 creates a highly proliferative phenotype, resistant to stress and apoptosis [25,26,27]. This supports rapid progression and metastatic behavior. Inhibition of miR-4286 results in a significant decrease in cell proliferation, suggesting that it maintains an active cell cycle by suppressing tumor suppressor genes. Upon miR-4286 suppression, markers of enhanced apoptosis are observed—caspase activation and an increase in Annexin V-positive cells—indicating that it stabilizes survival under stress [28]. Evidence also suggests that miR-4286 supports tumor cell adaptation to metabolic changes associated with oncogenic MAPK activation, including adjustments in glucose metabolism and oxidative stress [29]. miR-4286 can thus be considered a marker of high tumor activity and for dynamic monitoring of therapeutic response, particularly during progression under targeted therapy. Using NanoString technology, DiVincenzo et al. revealed different expression of miR-4286 in ulcerated primary cutaneous melanoma compared to nonulcerated tumors, as it was downregulated in the first ones [30]. The comparison was done only between these two types of melanoma but not in comparison to normal skin like in our study. In this regard, miR-4286 could be differently regulated in ulcerated melanoma and further evaluation of this observation is needed.

Both miR-146a-5p and miR-4286 represent important regulators that complement the oncogenic program driven by BRAFmut. The former acts as an immunological modulator, shaping a tumor-favorable low-inflammatory environment, while the latter enhances the proliferative and anti-apoptotic properties of melanoma [31,32,33,34,35]. Combined with MAPK signaling, these miRNAs form a stable oncogenic model that supports growth, progression, and immune evasion in BRAF V600E-positive tumors. This study demonstrates for the first time that miR-146a-5p and miR-4286 are both overexpressed in metastatic melanoma, especially those harboring the BRAF V600E mutation, highlighting their potential role as key regulators of tumor progression and immune evasion. Elucidating the key regulatory roles of miR-146a-5p and miR-4286 in melanoma opens a new field for integrated therapeutic strategies: simultaneous targeting of oncogenic signaling and modulation of tumor immune evasion. Although the current manuscript demonstrates upregulation of both miRNAs, critical appraisal of the literature suggests that miR-146a-5p retains more established biological significance, whereas the role of miR-4286 requires further functional investigation to be definitively identified as a key regulatory factor.

Integrative analysis using TargetLink 2.0 showed that miR-146a-5p and miR-4286 are associated with critical signaling networks, including TGF-β, NF-κB, and PTEN/PI3K/AKT, and that their combined action likely coordinates cytoskeletal remodeling, cell migration, and invasion. Furthermore, bioinformatic analyses identified WASF2 as a shared target of both miRNAs, highlighting their potential role in regulating cell motility and metastatic potential. WASF2 represents a convergence point between TGF-β, NF-κB, and PTEN-regulated signaling networks, integrating transcriptional, signaling, and cytoskeletal mechanisms linked to tumor progression and metastasis. This central role underscores the significance of WASF2 as a potential molecular mediator and therapeutic target, especially in aggressive and metastatic tumors, including BRAF-positive melanoma. Recently, lower WASF2 expression was observed in 11 different types of cancer, including skin and uveal melanoma and associated with resistance to chemotherapy for several GDSC (Genomics of Drug Sensitivity in Cancer) or CTRP (Cancer Therapeutics Response Portal) small molecules (https://www.aging-us.com/article/205993/text (accessed on 5 January 2026)).

Our findings not only underscore the central role of miR-146a-5p and miR-4286 in the biology of melanoma but also suggest opportunities for novel therapeutic strategies. Combining targeted therapy against BRAF and the MAPK/ERK pathway with modulation of these miRNAs could simultaneously suppress oncogenic signaling and reduce the immunosuppressive tumor microenvironment, thereby optimizing personalized treatment for patients with aggressive melanoma. Our study identifies miR-146-5p and miR-4286 as key regulators in metastatic melanoma, highlighting their potential as biomarkers and therapeutic targets with direct applicability in personalized and translational medicine.

In summary, this study provides the first evidence of the co-expression and functional association of miR-146a-5p and miR-4286 in metastatic melanoma. These miRNAs could represent both potential biomarkers and molecular targets for future integrated therapies, establishing a new paradigm in the management of aggressive melanoma. The limitation of our study is the modest cohort size, but the consistent trends observed provide a reliable indication of the upregulation of miR-146a-5p and miR-4286 and justify further investigation in larger cohorts. To further strengthen the reliability and robustness of our results, an independent validation of the most relevant miRNAs, specifically miR-146a-5p and miR-4286, using RT-qPCR should be done in future experiments.

Our study focuses on multiplex expression profiling and integrative bioinformatic modeling and establishes a systematic framework for future functional validation studies.

## Figures and Tables

**Table 1:** Summary of microRNA expression analysis for the two subgroups of analyzed melanoma.

Analysis	N miRNAs in BRAFwt Melanoma	N miRNAS in BRAFmut Melanoma
MicroRNAs in at least 1 sample	231	275
MicroRNAs in >50% of samples	122	155
MicroRNAs in all samples	39	49

**Table 2:** Average counts for overexpressed miRNA in the analyzed tumor and control samples.

	miR-146a-5p Counts	miR-4286 Counts	miR-4488 Counts	miR-1268a Counts
BRAFwt melanoma n = 8	98.19 (*p* < 0.04)	361.48 (*p* < 0.01)	315.95	155.69
BRAFmut melanoma n = 7	157.98 (*p* < 0.02)	581.89 (*p* < 0.03)	165.54	116.41
Control skin	22.19	57.12	77.23	52.8

## Data Availability

Detailed miRNA expression data and RT-qPCR results can be provided upon reasonable request to the corresponding author.
